# Quantifying western blots: none more black

**DOI:** 10.1186/s12915-016-0339-1

**Published:** 2016-12-28

**Authors:** Graham Bell

**Affiliations:** BMC Biology, London, UK

## Abstract

Western blotting is among the most common techniques used in molecular biology and a simple way of assessing the presence or absence of a protein. It is also commonly used to compare protein levels in different conditions or in different tissues. This article illustrates some of the easy ways to arrive at a false conclusion when trying to quantify protein levels from western blots.

## Commentary

**Fig. 1.**
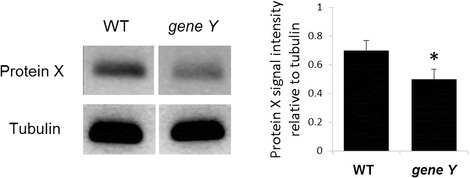
Expression levels of Protein X in wild type (WT) or *gene Y* mutant cells. One representative blot is shown of three independent experiments. *Bar chart* shows quantification of protein levels compared to tubulin control in each condition. *Error bars* show standard deviation, *P < 0.05

A group of biomedical researchers were looking for the effects on candidate downstream genes and proteins of knocking out Gene Y in a new mouse model. In their experiment, they assessed the reduction in the expression of Protein X in wild-type and *gene Y* mutant cells. They used tubulin levels as a control and quantified their results by calculating the band intensity of Protein X relative to tubulin—claiming a significant reduction of Protein X in the mutant (Fig. 1).

However, although the two tubulin controls look the same—and give the same intensity measurements using a simple image analysis tool—they do not represent the same underlying expression. In fact, the gel for the wild type was accidentally loaded with more of the sample. The chemiluminescent film was saturated, so the higher level of tubulin in the wild type was not reflected when the intensity measurements were taken: actually when the same amounts of sample were loaded, there was no change in expression of Protein X in the two conditions.

**Fig. 2.**
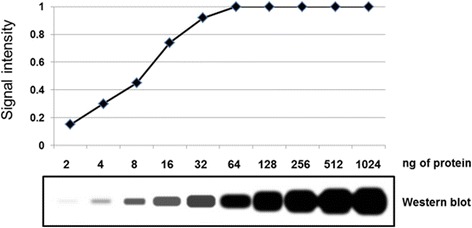
Dilution series western blotting experiment of increasing concentrations of Protein X (*bottom*). Graph shows quantification of relative levels of signal intensity (*top*). Note that concentrations of 64 ng and above each give an intensity value of 1, indicating that the bands are saturated in the view of the quantification software

This represents a general problem of quantifying western blots with simple image analysis software, which may be unable to discriminate between similar-looking bands that have fallen off the end of the linear scale. Figure 2 shows a stylised western blot of increasing concentrations of protein, and the “signal intensity” as measured by a commonly used software—in this example the last five concentrations gave the same intensity measurement despite representing very different amounts of protein.

**Fig. 3.**
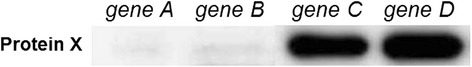
Western blot showing expression levels of Protein X in cells mutant for genes *A*, *B*, *C* and *D*, respectively

A related problem is how to represent very different protein levels on the same film (even if not trying to quantify the levels). Figure 3 shows the levels of Protein X in four samples (mutants for genes *A*, *B*, *C* and *D*, respectively). When the experimenters tried to extend the exposure time to make the bands in lanes 1 and 2 clearer, they accidentally over-exposed the bands in lanes 3 and 4, rendering their relative quantification analysis inappropriate. This can be a problem with using ‘housekeeping’ genes like actin or tubulin as the loading controls and a baseline for relative quantification, because these proteins tend to be expressed at higher levels than the target proteins: in order to measure the target protein, high amounts of the sample are loaded, saturating the housekeeping gene expression. (And in any case, the housekeeping gene expression may vary between tissues and experimental conditions, compromising its suitability as a control [1]).

Various methods have been proposed to overcome some of these problems, including using automated quantification platforms, using total protein stains as an alternative control, and optimising steps of the protocol, including protein loading, to ensure a linear dynamic range prior to detection [2, 3]. Complications arising from a lack of linearity in measuring techniques are not only found in western blot experiments of course, and are important to consider in other experimental systems—as the next “What is wrong with this picture?” article will explore.
